# Unlocking the potential of tumor‐derived DNA in urine for cancer detection: methodological challenges and opportunities

**DOI:** 10.1002/1878-0261.13628

**Published:** 2024-03-10

**Authors:** Birgit M. M. Wever, Renske D. M. Steenbergen

**Affiliations:** ^1^ Department of Pathology Amsterdam UMC, location Vrije Universiteit Amsterdam The Netherlands; ^2^ Imaging and Biomarkers Cancer Center Amsterdam The Netherlands

**Keywords:** biomarker, cancer, cfDNA, liquid biopsy, oncology, urine

## Abstract

High cancer mortality rates and the rising cancer burden worldwide drive the development of innovative methods in order to advance cancer diagnostics. Urine contains a viable source of tumor material and allows for self‐collection from home. Biomarker testing in this liquid biopsy represents a novel approach that is convenient for patients and can be effective in detecting cancer at a curable stage. Here, we set out to provide a detailed overview of the rationale behind urine‐based cancer detection, with a focus on non‐urological cancers, and its potential for cancer diagnostics. Moreover, evolving methodological challenges and untapped opportunities for urine biomarker testing are discussed, particularly emphasizing DNA methylation of tumor‐derived cell‐free DNA. We also provide future recommendations for technical advancements in urine‐based cancer detection and elaborate on potential mechanisms involved in the transrenal transport of cell‐free DNA.

## Graphical abstract

Urine is a rich source of biomarkers for cancer detection. Tumor‐derived material is released into the bloodstream and transported to the urine. Urine can easily be collected from individuals, allowing non‐invasive cancer detection. This review discusses the rationale behind urine‐based cancer detection and its potential for cancer diagnostics, particularly highlighting DNA methylation of tumor‐derived cell‐free DNA as a biomarker. Created with BioRender.com.

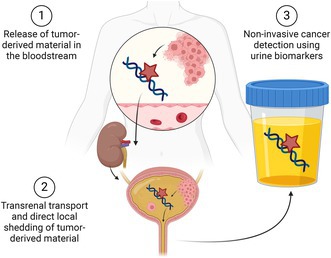

**Abbreviations**
cfDNA: cell‐free DNA
cfRNA: cell‐free RNA
EV: extracellular vesicle
FISH: fluorescent *in‐situ* hybridization
GAG: glycosaminoglycan
SAP: serum amyloid P component

## 1 Introduction

The global cancer burden of 19 million new cases in 2020 is expected to grow to over 30 million new cases in 2040, based on population growth and aging [1]. Cancer remains one of the leading causes of death worldwide, despite the development and use of innovative treatments during the last decades [2]. High mortality rates are partly caused by late diagnosis, as cancer is often detected at an advanced stage when treatment options are limited. Another major cause of high mortality rates is recurrence after effective initial treatment [3]. With the increased understanding of the underlying causes of cancer, and more cancer risk factors being known, also the number of individuals with a high cancer risk increases [4]. While this opens a window of opportunity for cancer prevention, it simultaneously places increasing pressure on the healthcare system, emphasizing the need for innovative cancer detection strategies. Urine‐based cancer detection methods could offer a potential solution to advance cancer diagnostics. This strategy would allow self‐collection from home and reduce the initial need to visit a healthcare professional, thereby alleviating the burden on both patients and healthcare systems.

Urine biomarkers have been extensively assessed for the improved diagnosis and management of bladder cancer, as comprehensively reviewed elsewhere [5, 6, 7]. This review discusses the rationale behind the use of urinary tumor‐derived DNA for detecting various non‐urological cancer types in urine to explore its potential in cancer diagnostics. In addition, we highlight evolving methodological challenges and untapped opportunities for tumor‐derived, DNA‐based biomarker testing in urine, particularly focusing on DNA methylation, and address their implications for future research. We also elaborate on potential biological mechanisms underlying the transfer of tumor‐derived DNA into the urine.

## 2 Liquid biopsy for cancer detection

Currently, invasive tissue biopsy procedures are performed at the diagnostic workup of individuals with suspected cancer. However, this procedure is often time‐consuming and associated with discomfort, high costs, and risk of complications. Moreover, cancerous lesions can be missed or incompletely captured during biopsy procedures. These limitations encourage the development of alternative and less invasive methods for cancer diagnosis [8]. The use of a liquid biopsy, which refers to the sampling and analysis of body fluids, holds potential as a novel tool for cancer detection and could complement tissue biopsy procedures in diagnostics [9, 10]. Blood is the most extensively investigated liquid biopsy, but also alternative fluids are actively pursued, including saliva, sputum, stool, urine, and vaginal fluid [11]. Their patient‐friendly collection method enables the convenient and repetitive acquisition of fresh tumor material, which can easily be performed at home.

Liquid biopsy is based on the release of tumor‐derived material, including cell‐free DNA (cfDNA), into circulation. Although the exact origin of cfDNA in the circulation is still under debate, proposed release mechanisms include apoptotic or necrotic cell death and active secretion [12]. In the past decades, there has been a steep increase in research on the molecular profiling of cfDNA derived from tumor cells, referred to as tumor‐derived cfDNA, for diagnostic purposes. Tumor‐derived cfDNA in circulation accurately reflects molecular alterations in the tumor tissue [13, 14] and has a high potential for non‐invasive cancer detection [15]. Molecular analysis of tumor‐derived cfDNA allows the identification of a variety of biomarkers, including, but not limited to, copy number changes, differences in fragment lengths, fusion genes, methylation, and mutations [16, 17].

The specific biological properties of cfDNA are related to its origin. The size of small cfDNA fragments in the circulation varies from 140 to 170 base pairs and peaks around 167 base pairs. This size is characteristic of the 147 base pairs of DNA wrapped around a histone protein, forming a mono‐nucleosome, and the presence of a linker DNA (20 base pairs) which protects DNA from degradation [18, 19, 20]. In healthy individuals, the majority of cfDNA is derived from leukocytes [21]. Generally, the amount of cfDNA in circulation is extremely low (0–100 ng·mL^−1^) but elevates under normal physiological conditions, such as exercise, and pathological conditions, such as inflammation, tissue trauma, and cancer [22]. The abundance of tumor‐derived cfDNA in the blood of cancer patients is described to be highly variable, ranging from 0.01 to 90% of the total cfDNA. The abundance of tumor‐derived cfDNA also differs per tumor type and stage, depending on tumor localization and vascularization [23, 24].

**Fig. 1:**
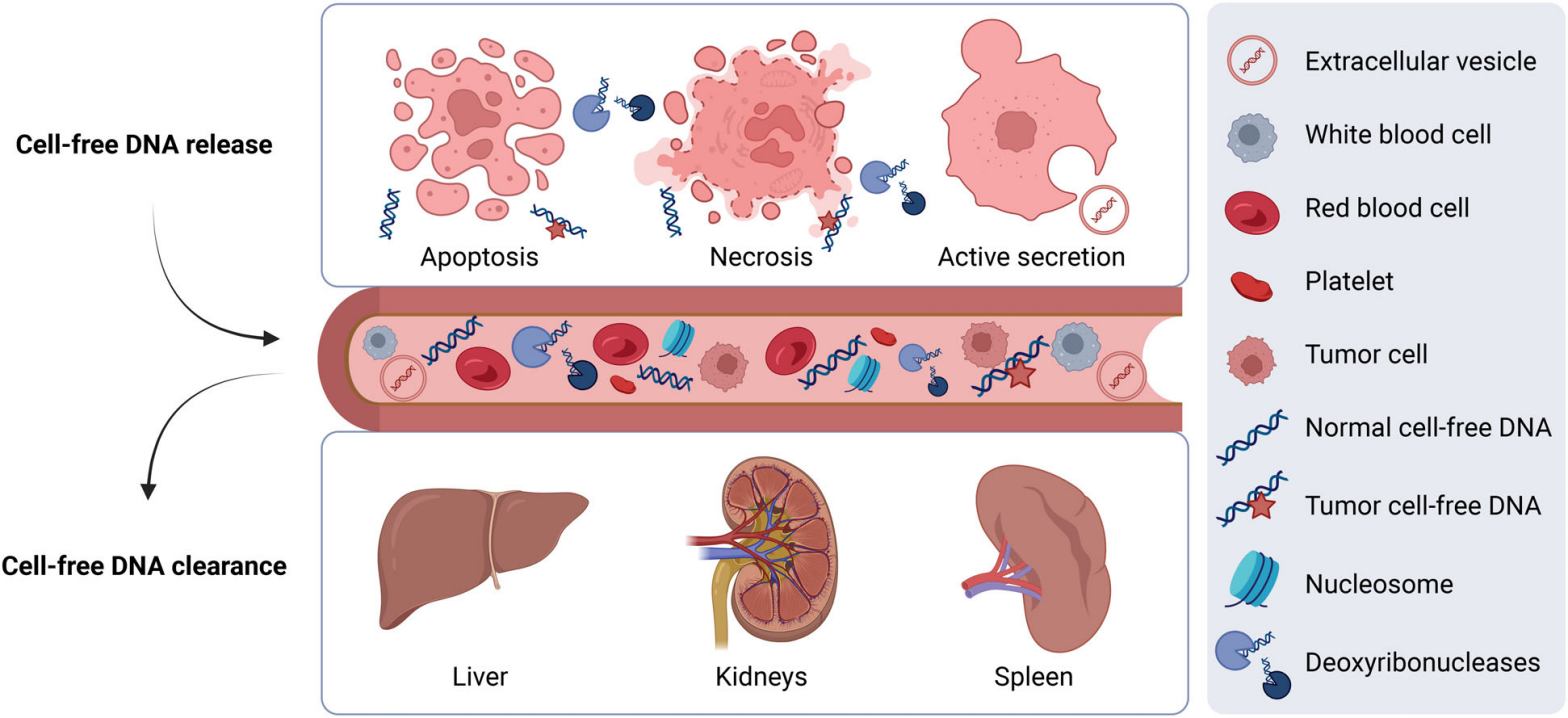
Cell‐free DNA release and clearance mechanisms. Tumor‐derived and normal cell‐free DNA is released into the bloodstream by cell death (apoptosis or necrosis) and active secretion. Nuclease digestion by deoxyribonucleases plays a role in both generating and clearing cell‐free DNA. Other routes of cell‐free DNA clearance involve absorption in the liver and spleen and transrenal excretion. Created with BioRender.com.

The presence of cfDNA is also largely determined by degradation and clearance rates [25]. The described half‐life of cfDNA ranges from 16 min to 2.5 h [26]. Enzymes that degrade DNA, known as deoxyribonucleases, play a role in both generating cfDNA during cell death and clearing cfDNA in the bloodstream [27]. During the last decades, several routes of cfDNA clearance have been proposed. Yet, the exact underlying mechanisms remain elusive as this complex process involves multiple filtering organs. It is thought that the majority (71–85%) of nucleosomes are removed from circulation by the liver [28], followed by transrenal glomerular excretion and absorption in the spleen [29, 30]. The cfDNA release and clearance mechanisms are summarized in Fig. 1.

Transrenal excretion of cfDNA was first described by Botezatu and colleagues in 2000 [31]. In this pioneer study, human DNA and radioactively labeled DNA were injected in mice and both were detected in the urine. They also described the presence of Y‐chromosomal DNA in the urine of women carrying a male fetus and women who were transfused with blood from a male donor. Furthermore, *KRAS* gene mutations were detected in the urine of patients diagnosed with colorectal cancer whose tissue biopsies showed the same mutations [31]. The presence of tumor‐derived DNA in the urine offers opportunities for urine‐based cancer detection [32].

## 3 Urine as a novel liquid biopsy

Urine is a relatively new liquid biopsy as compared to blood. Even though blood‐based cancer detection methods have shown high clinical potential, particularly for their use in diagnostics and treatment response monitoring [33, 34], this method comes with several drawbacks. Unless dried blood spots are used [35], the collection of blood requires in‐person visits and is usually performed by a specialist. Moreover, only limited amounts of blood can be collected per time point. Urine offers an attractive sample type for diagnostics, as it can be collected non‐invasively in large volumes, which allows for serial sampling at home with high patient acceptance [36].

Given the advantages of urine collection, urine‐based tests could address a wide range of clinical challenges in cancer management. Urine has primarily been explored as a liquid biopsy for urogenital cancer types that directly release DNA fragments into the urine, including bladder, cervical, endometrial, and prostate cancer [37, 38, 39, 40, 41, 42, 43, 44, 45, 46, 47, 48, 49]. Interestingly, transrenal DNA excretion has also been confirmed for cancer types in close proximity to the urethra, including bladder and cervical cancer [50]. However, in recent years, there has been growing interest in the detection of non‐urogenital cancers in urine in which direct shedding is unlikely, including colorectal, liver, lung, and ovarian cancer [51, 52, 53, 54, 55, 56, 57, 58, 59, 60, 61, 62, 63, 64], as also comprehensively reviewed elsewhere [11, 65, 66]. The transrenal excretion of tumor‐derived cfDNA into the urine offers opportunities for the detection of virtually any cancer type that releases cfDNA into the bloodstream [31].

**Fig. 2:**
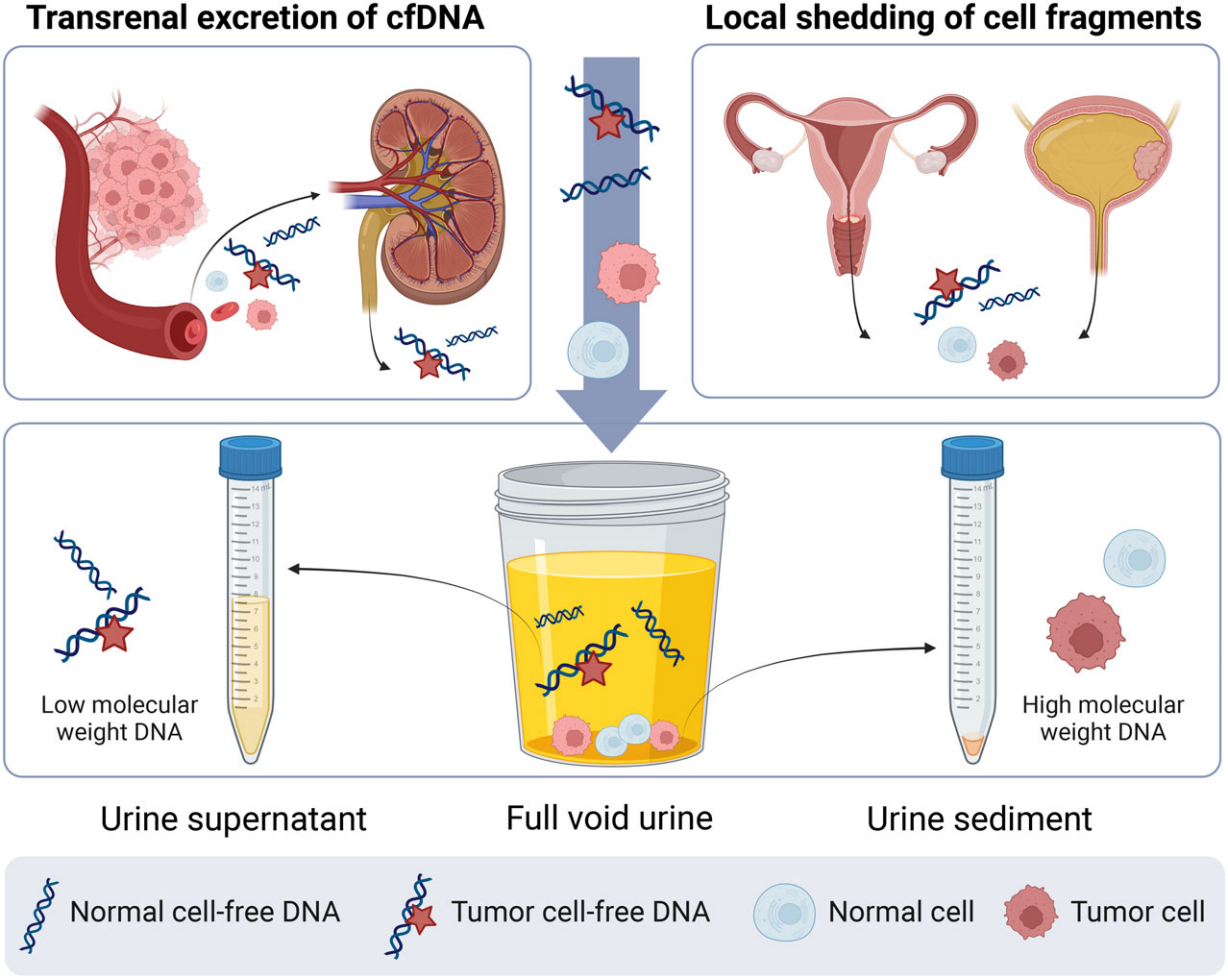
Urine as a liquid biopsy for cancer detection. Urine consists of different components, including normal and tumor‐derived cell‐free DNA and cellular debris from both normal and tumor cells. Cell‐free DNA is transported into the urine by transrenal excretion. The anatomical position of the cervix and bladder allows the local shedding of cells and DNA into the urine. Created with BioRender.com.

Urine is a dynamic fluid that consists of a variety of components (Fig. 2). DNA found in the urine can be broadly classified into a high and low molecular weight group. The high molecular weight DNA (≥ 1 kb in size) is derived from cellular debris, such as immune cells and exfoliated cells from the genital tract or distal urethra [65]. The low molecular weight DNA (10–250 bp in size) comprises small transrenally excreted fragments [67]. Full void urine contains both high and low molecular weight DNA, which can be divided by separating the urine into two fractions: the urine sediment and the urine supernatant. The urine supernatant is enriched for cfDNA (low molecular weight DNA), while the urine sediment mostly contains cellular debris (high molecular weight DNA). Tumor signals may originate from both low and high molecular weight DNA, as shown by the presence of increased methylation levels of cancer‐associated genes in all urine fractions for bladder, cervical, and endometrial cancer [46, 47, 68].

The optimal urine fraction for cancer detection may depend on the location of the tumor. For example, the urine sediment was shown to be optimal for detecting bladder and cervical cancer, as this fraction most likely contains the highest yield of exfoliated cancer cells [41, 68]. Urine supernatant, on the other hand, appeared most suitable for colorectal cancer detection [52]. The standardization of pre‐analytics is key for obtaining reliable and reproducible measurements as extensively reviewed elsewhere [66].

The concentrations of tumor‐derived nucleic acids in liquid biopsies, including urine, can be extremely low in some cases, depending on disease stage, cancer type, and treatment [69]. Total tumor‐derived cfDNA levels can be particularly low in patients with early‐stage disease or patients diagnosed with poorly shedding cancer types, such as renal cancer or glioma [70, 71]. Treatment with senescence‐inducing cytotoxic therapies may also reduce the release of tumor DNA [72]. The tumor‐derived cfDNA fraction in urine is often further diluted by the large volumes of voided urine and the presence of non‐tumor‐derived DNA. Nucleic acids in the urine predominantly originate from non‐malignant cells, considering the presence of intact urinary tract and inflammatory cells. Moreover, the continuous release of cfDNA from virtually any tissue type, coupled with its translocation into the urine, further contributes to the heterogeneous nucleic acid composition in urine [73, 74]. The low levels of tumor‐derived cfDNA in the urine call for optimal DNA extraction methods, cancer‐specific biomarkers, and sensitive biomarker detection techniques.

## 4 Maximizing the urine cell‐free DNA yield

The low cfDNA recovery rate from urine poses challenges for downstream DNA‐based analyses. Even though urine can be collected in large volumes, most existing isolation methods are unable to process large urine volumes, with a maximum urine processing volume of 40 mL using the Quick‐DNA Urine kit (Zymo Research, Irvine, CA, USA). The urine input volume could be increased by implementing a urine concentration step into the workflow before DNA isolation [73]. Reckamp and colleagues concentrated large urine volumes up to 100 mL to only 4 mL before isolation using Vivacell 100 concentrators (Sartorius, US) for *EGFR* mutation detection in the urine of non‐small cell lung cancer patients. Both sensitivity and specificity increased when using larger urine volumes (90–100 mL vs. 10–89 mL) [59]. Yet, whether increasing the urine volume also enhances test accuracy remains understudied. Alternatively, pooling urine samples collected at different time points might be more effective in capturing the tumor signal, as shown previously for the detection of non‐small cell lung cancer in urine [75].

Collecting a particular urine portion, such as the initial urine stream (also known as the first void), poses another strategy to improve test performance, as reported for high‐risk human papillomavirus DNA testing [76]. Designated collection devices, such as the Colli‐Pee® device (Novosanis, Belgium), could ease and standardize the collection of a particular urine portion from home. For applications relying on transrenally excreted cfDNA, such as colorectal or lung cancer detection in urine [52, 58, 59, 61], the collection of the midstream urine might be preferred, as this usually contains less mucus and exfoliated cell debris.

The use of an optimal cfDNA extraction kit could also maximize the urine cfDNA yield and thereby boost test performance. The origin of liquid biopsy, being a plasma or urine sample, affects both the recovery rate and minimal length of cfDNA detected. Urine samples generally tend to contain shorter fragments due to glomerular filtration and increased DNA degradation [77]. Median fragment sizes are estimated at 133 base pairs in urine, as compared to ~ 166–169 base pairs in plasma. Tumor‐derived fragments are most likely shorter with a size of 101 base pairs in urine, as compared to ~ 133–160 base pairs in plasma [78, 79].

Keeping in mind the relatively high fragmentation and low concentration of cfDNA in urine, an ideal isolation kit should be capable of recovering short DNA molecules from samples with highly diluted concentrations of DNA. The results of three studies comparing commercially available urinary cfDNA isolation kits are summarized in Table 1 [77, 80, 81]. Within these studies, a total of five kits were examined, including the MagMAX Cell‐Free DNA Isolation Kit (Thermo Fisher Scientific, Waltham, MA, USA), Urine Cell‐Free Circulating DNA Purification Midi Kit (Norgen Biotek, Thorold, ON, Canada), QIAamp Circulating Nucleic Acid Kit (Qiagen, Hilden, Germany), NextPrep‐Mag Urine cfDNA Isolation Kit (PerkinElmer, Waltham, MA, USA) and Quick‐DNA Urine Kit (Zymo Research). Notably, the number of included urine samples in such studies is relatively small, ranging from 5 to 10. Fragment sizes were examined by real‐time quantitative PCR or electrophoresis systems (Agilent, Amstelveen, The Netherlands). Out of the five kits evaluated in these studies, none of them showed particularly outstanding results in all characteristics and advantages at the same time. Generally, magnetic bead‐based extraction kits allow for automation and are therefore not as hands‐on‐demanding as compared to column‐based kits. Yet, the column‐based Norgen kit enabled the recovery of the shortest fragments of only 25 base pairs [77]. The Zymo kit permits the largest urine starting volumes at the most affordable price but also extracts large cellular DNA fragments besides cfDNA [80]. The outcomes of these comparison studies underline that the optimal extraction method is yet to be defined and may also depend on the downstream application.

**Table 1:** Comparison of five commercially available urinary DNA extraction kits. Kits are compared according to the extraction strategy, urine input volume and minimally extracted fragment size, and price. bp, base pairs.

Name/manufacturer	MagMAX	Norgen	QIAamp	PerkinElmer	Zymo
Extraction strategy	Magnetic beads	Column	Column	Magnetic beads	Column
Input volume (mL)	0.5–10	2–10	1–5	4–20	≤ 40
Minimum size (bp)	80	25	150	100^a^	100^b^
List price/extraction (€)	11.0	25.3	24.6	12.2	10.8
Citation	[77, 80]	[77, 80, 81]	[77, 80]	[81]	[80]

^a^ Minimal effective size detection has not been tested below 100 bp [81].

^b^ Based on official website [82].

## 5 DNA methylation as biomarker for urine‐based cancer detection

One of the well‐studied cancer‐associated epigenetic changes to be used as biomarker for cancer detection is DNA methylation [83]. Cancer cells exhibit abnormal methylation patterns, which can drive malignant transformation. Global hypomethylation contributes to genomic instability [84], while focal hypermethylation at gene promoters leads to the silencing of genes involved in tumor suppression [83]. DNA methylation changes are already observed during the earliest phases of carcinogenesis and are therefore attractive for early cancer detection [83, 84]. It has been shown that DNA methylation markers even allow the detection of precancerous lesions of the anus [85], cervix [42], colon [86], oral cavity [87], and vulva [88]. Methylated DNA remains stable after long‐term storage of clinical samples and can be analyzed efficiently using relatively inexpensive methods [89, 90]. Methylation analysis does not require the presence of intact tumor cells for interpretation and can also be performed on fragmented DNA [89]. This offers opportunities for methylation testing on fragmented tumor‐derived DNA in the urine for the detection of cancer types in less proximity to the bladder and urethra. Urine‐based DNA methylation testing has successfully been performed for the detection of a variety of non‐urological cancer types, including cervical [41, 42, 46, 55], colorectal [51, 52, 60, 63], endometrial [47, 48], liver [54], lung [58, 61, 75], and ovarian [62, 91] cancer. In all studies, methylation status was assessed by PCR‐based methods and urine‐based methylation testing was mostly evaluated for diagnostic purposes.

However, despite clear advantages, DNA methylation marker testing in urine faces several challenges, including (a) the identification of cancer‐type‐specific methylation markers and (b) methylation changes in non‐cancerous conditions. Biomarkers that allow the detection of multiple cancer types are known as pan‐cancer markers. For example, *CDO1* gene methylation has been described as biomarker for the detection of endometrial, lung, and ovarian cancer detection in urine [48, 58, 61, 62]. From an implementation perspective, pan‐cancer markers might be most applicable in a targeted setting to rule out malignancies in a certain high‐risk group. When implemented into a screening setting, cancer localization after detection forms a critical test element. To this end, pan‐cancer methylation markers could potentially be combined with tissue‐specific methylation markers to determine the primary tumor site [92, 93, 94]. Methylation levels can also be elevated due to non‐cancerous conditions, such as aging and smoking, potentially leading to false‐positive results [95, 96, 97]. The strong correlation between non‐cancerous conditions and changes in DNA methylation underlines the importance of including matched control groups [95]. Moreover, knowledge of changes in DNA methylation levels in the urine of individuals with benign conditions is essential to ensure a high specificity for cancer detection in a clinical setting.

## 6 Technical considerations to enhance methylation test accuracy in urine

Several technical optimizations could be considered to enhance methylation test accuracy in urine. From a practical perspective, the use of targeted PCR‐based methylation assays, rather than epigenome‐wide signatures, is currently still the method of choice in a diagnostic setting because of its relatively low costs and ease of implementation. The careful selection of the targeted genomic location and design of PCR‐based assays are crucial to obtain clinically meaningful results. Moreover, selective enrichment of methylated DNA could improve methylation detection.

### 6.1 Selecting a clinically relevant differentially methylated region

The genomic location of the targeted PCR assay is crucial for the development of clinically relevant methylation assays. Genomic locations of methylation markers for urine‐based cancer detection can be derived from methylation profiling of other sample types, like tissue, cervical scrapes, or self‐samples, as proven suitable in previous studies on bladder, cervical, colorectal, endometrial, and lung cancer [41, 48, 52, 58, 98, 99, 100, 101]. However, one may expect that employing a different sample type would require a different set of markers due to variations in the presence of background DNA and generally lower levels of tumor‐derived DNA. Therefore, it would be interesting to investigate whether alternative DNA methylation markers suitable for cancer detection in urine could be discovered by methylation sequencing of urine cfDNA.

Bisulfite sequencing for the discovery of differentially methylated regions in urine samples can be technically challenging due to low amounts of fragmented cfDNA and DNA degradation during sodium bisulfite treatment [102, 103]. The use of bisulfite‐free sequencing approaches using immunoprecipitation‐based enrichment of methylated cfDNA, such as methylated cfDNA sequencing (MeD‐Seq) [104], or cytosine conversion based on enzymatic processes, such as enzymatic methyl sequencing (EM‐Seq) [105], could overcome this issue. EM‐Seq has successfully been performed previously using minute amounts of urinary tumor‐derived DNA of ovarian cancer patients [62]. Alternatively, long‐read sequencing, such as Oxford Nanopore Technologies, could be utilized for DNA methylation analysis at a single‐base, single‐molecule resolution, by exploiting the differences in electric current of methylated and unmethylated cytosines [106]. Although long‐read sequencing was successfully performed in urine of bladder cancer patients [107], a remaining technical challenge is the high DNA input needed, which might be particularly challenging for early‐stage cancers with a low quantity of tumor‐derived DNA in the urine.

### 6.2 Revisiting PCR‐based methylation assay design

Instead of looking for alternative methylation markers, test accuracy of PCR‐based methylation assays could be improved by revisiting its design. The primer and probe design quality is crucial for obtaining reproducible methylation level measurements and often underlies the differences observed across studies investigating the same methylation marker [108, 109]. For example, designing assays with an amplicon length below 120 base pairs is crucial for the amplification of short urinary cfDNA fragments. Other considerations for optimal quantitative methylation‐specific PCR assay design for liquid biopsies are outlined in Table 2 and stem from both expert knowledge of the authors and relevant literature [109, 110].

**Table 2:** Considerations for designing optimal multiplex quantitative methylation‐specific PCR assays for liquid biopsies. Partially adapted from Massen [109] and Snellenberg [110]. LNA, locked nuclear acid; MGB, minor groove binding; Tm, melting temperature.

Location selection	Select a clinically relevant differentially methylated region, based on literature, whole‐genome sequencing data, or publicly available datasets (e.g. via mexpress.be [111]). Select a region with a suitable density of CpG sites. Avoid using regions with three or more consecutive CGs.
Primer and probe design	Aim for an amplicon size between 60 and 120 base pairs. Design primers and probes with at least two methylated Cs each. Design primers with a potentially methylated C at the 3′‐end. Design primers with a Tm between 58 and 63 °C. Design forward and reverse primer with ≤ 2 °C difference. Design probe with a Tm of 10 °C higher than primer Tm. Avoid probe designs with a G at the 5′‐end. Aim for a GC content between 30% and 80%. Check target specificity of primers using blast. Test amplification of the desired amplicon *in silico*. Check formation of hairpins, self‐dimers, and cross‐dimers *in silico*. Use LNAs and/or MGB probes to increase the Tm of short primers and probes.
Multiplex design	Design primers and probes of different targets with a similar Tm. Check cross‐hybridization of primers and probes of different targets *in silico*. Select non‐overlapping fluorescent labels for different targets.
Primer and probe testing	Validate amplification of the correct product by gel electrophoresis. Determine optimal primer Tm using gradient PCR reactions. Ensure PCR efficiencies are close to 100% (range 80–110%) and *R* ^2^ > 0.98. Refine primer and probe concentrations using primer and probe limiting assays. Determine optimal cutoff and baseline values per target.

### 6.3 Selective enrichment of methylated DNA

Methylated DNA can also be selectively enriched before PCR or sequencing by leveraging the specific properties of 5‐methylcytosine. This could be accomplished by using affinity approaches that specifically bind to methylated DNA, using either 5‐methylcytosine antibodies (e.g. cell‐free methylated DNA immunoprecipitation‐sequencing [cfMeDIP‐seq] [112]), methyl‐CpG binding domain proteins (e.g. methyl‐binding domain sequencing [MBD‐seq] [113] or methylated DNA capture by affinity purification [MethylCap‐seq] [114]). Alternatively, methylation‐dependent restriction enzymes could be used to enrich for methylated DNA (e.g. [MeD‐seq] [104]). Methyl‐binding proteins can also be immobilized on a capture surface to selectively enrich for methylated DNA from liquid biopsy samples [115].

## 7 Alternative urine biomarkers

The low abundance of methylation signals in urine may limit the detection of cancer, particularly in the case of early‐stage cancers. Therefore, technologies and expertise from the more advanced plasma‐based cancer detection field could be leveraged to explore a broader range of biomarkers in urine. In addition to DNA methylation signatures, tumor‐derived cfDNA in the urine contains information about various other genetic and epigenetic events. Moreover, urine is a rich source of biomarkers beyond DNA, including circulating RNA, proteins, metabolites, and exfoliated tumor cells.

### 7.1 Concurrent analysis of genetic and epigenetic events

Recent advances in sequencing technologies and bioinformatics analyses unlock the potential of retrieving genetic and epigenetic changes from the same sample and could most likely also be applied to urine samples. The recently developed MethylSaferSeqS technology allows a concurrent analysis of copy number aberrations, mutations, and methylation signals from the same cfDNA molecules [116]. This innovative approach separates the original DNA strands from the copied DNA strands during library preparation, which enables the analysis of epigenetic alterations in the original strand and the recognition of genetic alterations (including cytosine‐to‐thymine mutations) in the copied strand. The simultaneous analysis of genetic and epigenetic markers could add sensitivity to cancer detection methods [117, 118]. Another advanced sequencing platform for assessing genetic and epigenetic information from the same strand is known as Five/Six‐letter‐seq [119]. This approach is based on enzymatic conversion and also allows the recognition of both 5‐methylcytosine and 5‐hydroxymethylcytosine, besides the four regular nucleotides. DNA hydroxymethylation signatures in cfDNA appeared additive to DNA methylation signatures, as shown for the detection of colorectal and pancreatic cancer in plasma [120, 121]. The development of novel profiling methods to accurately capture and analyze hydroxymethylation markers could further enhance its utility for cancer detection.

### 7.2 Cell‐free DNA fragmentation patterns

In both plasma and urine, circulating cfDNA predominantly exists in a bound state to histone proteins, since naked DNA is quickly degraded by DNA nucleases [122]. The structural properties of cleaved cfDNA, such as fragment size distributions or fragment‐end sequences, are non‐random and associated with cancer [69, 123, 124]. Characteristic fragmentation patterns can be measured in liquid biopsies [69], including urine [61, 117, 125, 126, 127]. The analysis of fragmentation patterns allows the detection of a wide variety of cancer types within a single assay, as shown in plasma using the ‘DNA evaluation of fragments for early interception’ (DELFI) approach [128]. Moreover, the tissue of origin can be identified by inferring nucleosome positioning near transcription start sites from the start and end points of cfDNA fragments [129]. Using conventional short‐read sequencing, typically shorter fragments are found to be more abundant in the urine of cancer patients as compared to controls [125, 126]. Nevertheless, this area of research continues to be actively explored as long‐read sequencing revealed that a similar tumor fraction was found in larger fragments of over 300 base pairs [107].

### 7.3 Circulating RNA

Circulating RNA provides another promising biomarker class for cancer detection in liquid biopsies. Different types of circulating RNA include messenger RNAs, microRNAs, long non‐coding RNAs, and circular RNAs. RNA molecules are typically stable due to their structural characteristics and/or the protection provided by enrichment in vesicles, such as exosomes [130, 131]. For prostate cancer, assays for the measurement of a 2‐gene RNA expression panel [132] and exosome RNA expression signatures [133] in urine collected after rectal examination are readily used along with standard‐of‐care, in both a diagnostic and prognostic setting. The value of circulating microRNAs as cancer biomarkers in urine has been described for several cancer types, including endometrial [134, 135] and ovarian [135] cancer. Urine‐derived expression profiles of long non‐coding RNAs and circular RNAs are explored for the detection of urological cancers but remain limitedly investigated for non‐urological cancer types [130, 136, 137].

The integration of DNA and RNA analyses from a single urine sample could be facilitated by using the urine sediment for methylation measurements and the urine supernatant for RNA analysis, as shown previously for prostate cancer detection [138]. Alternatively, cell‐free RNA (cfRNA) and cfDNA could be extracted simultaneously from a single sample and subsequently be divided for independent cfRNA and cfDNA sequencing analyses. Direct comparisons have shown that cfRNA provides a more sensitive measurement of certain biomarkers, including mutations and fusion genes [139].

### 7.4 Circulating proteins

The combination of protein biomarkers with nucleic acid signatures could also improve test sensitivity by leveraging their complementarity. An example of a blood‐based multi‐cancer detection test relying on the presence of both cancer‐specific mutations and overexpressed protein levels is the CancerSEEK test [140]. The urine proteome is considered to be less complex compared to the blood proteome, due to glomerular reabsorption of most proteins. Urinary protein biomarkers have been explored for the detection of varying cancer types [141], including endometrial [142, 143, 144], lung [145], and ovarian [143, 146] cancer. One potential drawback is the dynamic nature of protein concentrations in urine, which may present comparable challenges to those encountered with urine cfDNA [141].

### 7.5 Circulating metabolites

Changes in metabolites that reflect reprogramming of cellular metabolism, a core hallmark of cancer, are also attractive biomarkers beyond the (epi)genomic landscape [147]. Glycosaminoglycans (GAGs) are involved in cancer development and represent a promising class of tumor metabolism biomarkers. The analysis of GAGs may add sensitivity to existing cancer detection methods based on cfDNA. The accurate quantification of GAG profiles from both plasma and urine appeared valuable in detecting multiple cancer types, even those at stage I, as well as cancer types that release minimal amounts of cfDNA, such as renal cancer [148, 149]. The study on 580 urine samples reported a specificity of 62% at a predefined sensitivity of 95% for the detection of stage I and low‐grade cancers using GAG profiles [148].

### 7.6 Exfoliated tumor cells

Urine cytology offers an easily applicable and affordable method to detect cancer types that are known to exfoliate cancerous cells into the lower genital tract, such as bladder or endometrial cancer [45]. The routine collection and cytological examination of urine for bladder cancer detection is an established method in diagnostic laboratories [150]. Clinically adopted tests to improve the low sensitivity of cytology for bladder cancer detection include fluorescent *in‐situ* hybridization (FISH) and fluorescent immunocytochemistry‐based assays [151, 152]. Similar assays might also broaden the use of exfoliated endometrial cancer cells in the urine. The in‐depth molecular characterization of exfoliated tumor cells in the urine using sequencing methods, such as single‐cell sequencing [153], could also further increase the clinical utility of exfoliated tumor cells.

Taken together, integrating different biomarker classes poses a promising approach to improving the performance of urine‐based cancer detection methods. Combining multiple biomarker signatures retrieved from large datasets requires complex computational methods, such as machine learning algorithms or neural networks. Large patient cohorts are required to accurately train and validate multi‐dimensional molecular classifiers for cancer detection [69]. Moreover, standardization of pre‐analytics is crucial for accurate and reliable biomarker validation, as extensively reviewed elsewhere [66], and should be streamlined across different centers [154].

## 8 Unraveling the origin of urine cell‐free DNA

The urgency to implement liquid biopsy tests in the clinic may divert attention away from understanding the origin of cfDNA. Yet, enhancing our understanding of cfDNA biology and the origin of urine cfDNA is essential to improve its future clinical utility [74, 155, 156]. During the last decades, several hypotheses on the glomerular filtration of cfDNA have been proposed, including passive filtration through pores or active vesicle‐mediated transport [157, 158].

### 8.1 Transrenal transport of cell‐free DNA by passive filtration

Passive filtration may occur for the clearing of short cfDNA fragments through the glomerular filtration barrier [157]. However, circulating cfDNA fragments in both blood and urine are typically histone‐bound [122] with a molecular weight of around 200 kilodaltons (kDa) [159]. Under normal conditions, proteins with a molecular weight above 70–80 kDa are hindered from passing through the glomerular filtration barrier due to their size [160]. The glomerular membrane permeability reduces further for negatively charged molecules because of the negatively charged characteristics of the glomerular basement membrane itself [161]. Alternatively, the concept of protein filtration through larger shunt‐like pores has been described [162]. However, the prevalence of such pores is either extremely low or they may even be nonexistent according to the most recent literature [163].

Given this information, it is unlikely that nucleosomes pass through the glomerular membrane in their typical configuration. In the bloodstream, nucleosomes are protected from nuclease digestion and rendered soluble due to the substitution of histone H1 with the serum amyloid P component (SAP) [164]. Although hypothetical, it is possible that binding to SAP mediates nucleosome unwinding which might in turn facilitate glomerular passing [158]. An intriguing finding is that the tissue amyloid P component is naturally present on the glomerular basement membrane, supporting its potential role in nucleosome clearance [165].

### 8.2 Transrenal transport of cell‐free DNA by extracellular vesicles

Another likely scenario of transrenal passing of cfDNA is through extracellular vesicles (EVs), of which exosomes are most intensively studied. EVs play a key role in intercellular communication and are involved in a wide variety of physiological and pathological processes, including cancer [166]. The lipid bilayer surrounding EVs acts as a protective layer against nucleases and immune cells [167]. It has been described that EVs carry nucleic acids, including cfDNA [168]. However, the precise localization of nucleic acids in EVs is still under debate (i.e. enclosed within the lumen or surface‐bound) [155, 169]. Localization might also be affected by physiological factors, as it has been shown that exercise specifically increased exosomal DNA levels on their surface [170]. The average fragment size of exosome‐derived DNA appeared longer as compared to the classical size of cfDNA, with fragment sizes up to 4000 base pairs [171, 172]. This could explain the presence of long DNA fragments in the urine supernatant fraction [127].

Urine comprises different types of EVs, which are mostly derived from the urinary tract but may also originate from more distant anatomical sites [173]. It is currently unknown how EVs are transported from the blood to the urine. Exosomes, which are amongst the smallest EVs, have a size range from 40 to 100 nm. Therefore, translocation through glomerular pores seems unlikely, as only vesicles with a size range of 6 to 8 nm can pass [174]. In a diseased state, disruptions of the membrane pores might occur which could potentially facilitate the passing of larger vesicles [175]. Alternatively, vesicles could enter the urine by being absorbed by the proximal tubule cells or podocytes via selective endocytosis [176, 177].

### 8.3 Tubular reabsorption of cell‐free DNA after glomerular filtration

After glomerular filtration, tubular reabsorption occurs in which various substances are transported back into the bloodstream to regulate the urine composition [178]. The potential reabsorption of cfDNA after filtration might be a crucial determinant of total cfDNA levels in the urine. The biological functions of cfDNA molecules, including their immunogenic effects and role in cellular homeostasis [74, 155], provide reasons for potential reabsorption after filtration. Moreover, efficient recycling of circulating nucleotides could conserve energy and resources within the body and help to maintain a balance between DNA synthesis and degradation. From an energy‐conserving perspective, it is most likely that the reabsorption of cfDNA predominantly occurs via passive mechanisms.

Presently, biological mechanisms underlying transrenal transport of cfDNA remain largely underexplored and warrant deeper investigation. A more in‐depth understanding of transrenal clearance of cfDNA could guide the optimization of sampling procedures and ensure that urine‐based tests yield clinically meaningful results, which both represent vital factors for the successful translation of urine‐based tests into clinical practice.

## 9 Conclusions

The diagnostic potential of urine for cancer detection is clear. However, it is essential to underline that this field faces challenges that must be overcome before its translation into clinical practice, including the low levels of tumor‐derived DNA in the urine, the search for cancer and cancer‐type‐specific biomarkers and the validation of these biomarkers across diverse source populations. These hurdles could be addressed by technological advancements that facilitate enhanced biomarker detection in urine. Moreover, this review underlines the untapped opportunities for urine biomarker testing beyond tumor‐derived DNA. Continued development of such approaches opens avenues for effective and patient‐friendly cancer detection with broad applicability in future clinical practice.

## Conflict of interest

RDMS has a minority share in Self‐screen B.V., a spin‐off company of Amsterdam UMC, location VUmc. Self‐screen B.V. holds patents and products related to the work (biomarkers for cancer detection). BMMW declares no potential conflicts of interest. No writing assistance was utilized in the production of this manuscript.

## Author contributions

BMMW and RDMS conceptualized the study. BMMW conducted the literature review and generated the figures. BMMW and RDMS drafted the manuscript. Both authors approved the final version of the manuscript.
